# Enhancing Antioxidant and Cytotoxic Properties of CeO_2_ Through Silver Decoration: A Study on Ag@CeO_2_ Nanocomposites

**DOI:** 10.3390/nano15100748

**Published:** 2025-05-16

**Authors:** Chen Yang, Jingjing He, Siying Chen, Qiuping Li, Xuexia Lin

**Affiliations:** 1 Zhongshan Hospital (Xiamen), Fudan University, Xiamen 361015, China; 2 Xiamen Clinical Research Center for Cancer Therapy, Zhongshan Hospital, Fudan University, Shanghai 361015, China; 3 HQU Research Center for Emerging Pollutants Analysis and Ecotoxicology Assessment, College of Materials Science and Engineering, Huaqiao University, Xiamen 361021, China; 4 College of Chemistry, Huazhong Agricultural University, Wuhan 430070, China; 5 Zhongshan Hospital, Fudan University, Shanghai 200032, China

**Keywords:** Ag@CeO_2_, cytotoxicity, antioxidant activity, stability, electrochemical therapy

## Abstract

In this study, silver-decorated cerium oxide (Ag@CeO_2_) nanoparticles were synthesized through a simple mixing and pyrolysis process to enhance their antioxidant and antitumor properties. The synthesis methods for Ag@CeO_2_ were optimized, resulting in nanoparticles with varying antioxidant activities. Notably, the embedding of Ag nanoparticles within CeO_2_ during pyrolysis significantly improved the antioxidant activity and stability of the nanoparticles, leading to enhanced antitumor effects. The Ag@CeO_2_ nanoparticles exhibited strong cytotoxicity against tumor cells (MCF-7) while maintaining low toxicity toward normal cells (HUVECs). These properties, combined with their ability to modulate mitochondrial membrane potential, position Ag@CeO_2_ as a promising candidate for electrochemical therapy (ECT). This study highlights the antioxidant potential and the potential of Ag@CeO_2_ nanoparticles in tumor treatment and provides new insights into the application of nanomaterials in ECT.

## 1. Introduction

Over the past years, the field of precision medicine has been developing rapidly, promising to offer patients more personalized and effective treatment plans and bringing them a healthier and longer life. The side effects associated with traditional chemotherapy for tumor treatment are considerable, as these therapies often target and inhibit tumor cells while simultaneously inflicting toxic effects on normal cells and tissues. Due to individual patient variability, sensitivity to these drugs can differ significantly; those who exhibit heightened sensitivity are at an increased risk of experiencing severe adverse reactions or unpredictable health conditions. Given these limits, electrochemotherapy (ECT), as an effective local therapy for various tumors, has emerged as one of the most powerful tools of antitumor therapy [1,2,3]. ECT enables timely facilitation of chemotherapeutic drug delivery into special tumor cells, which promises to offer patients more personalized and effective treatment and bringing them a healthier and longer life [4,5]. However, it is challenging to develop a highly effective approach to minimize drug-related side effects while allowing for the controlled release of drugs according to the progress of disease or treatment.

Nanomaterials have garnered increasing attention in antitumor research due to their unique pharmacological properties [6,7]. These nanomaterials can serve as therapeutic agents for tumor treatment or as drug delivery vehicles capable of transporting anti-cancer medications directly to the tumor site while facilitating controlled release mechanisms. Besides, they possess high specific surface areas and substantial drug-carrying capacity. Their external surfaces and internal pores can be functionally modified. Moreover, they allow for controlled drug release that can be adjusted by varying conditions such as pH levels and temperature [8,9]. Given these advantages, the application of nanomaterials can enable minimization of drug-related side effects while ensuring targeted disease management. Gao et al. reported on a mesoporous calcium silicate-based nanocomposite designed for breast cancer therapy that effectively kills breast cancer cells without exhibiting cytotoxicity [6]. Jung et al. developed a novel grapefruit-inspired polymeric capsule (GPC) as a advanced nanomaterial carrier for energy storage, drug delivery, catalysis, and environmental application [7]. The GPC shell can prevent nanomaterial leakage and the influx of suspended solids, while its internal framework enhances structural stability and mass transfer rates. Tiron group synthesized a Mn^2+^ doped N-hydroxyphthalimide-derived carbon dots (Mn-CDs-NHF) composite nanomaterial for cell biology studies and showed that the Mn-CDs-NHF composite nanomaterial causes almost no damage to the cell viability of normal cells and can effectively reduce the volume of breast tumors while acting as a magnetic resonance imaging agent [10].

CeO_2_, as a metallic nanomaterial, possesses numerous unique properties including biocompatibility, chemical stability, and photocatalytic capabilities [11,12]. These attributes have facilitated its growing applications in drug delivery, bioimaging, photoablation therapy, and biosensors [13,14,15,16]. Furthermore, the accumulation of CeO_2_ at tumor sites has demonstrated certain antitumor characteristics for precisely targeting tumor tissues treatment [17,18]. Moreover, the synergistic effects of various nanomaterials or composite nanomaterials often yield enhanced biological properties. Loosen and his co-workers explored the feasibility of Ce/Zr-UiO-66 nanomaterials functioning as nano-enzymes and experimentally demonstrated that the concentration of Ce^4+^ directly influences both peptide bond hydrolysis and cysteine residue oxidation with catalytic activity [19]. Sun et al. investigated the ratio of Ce^3+^/Ce composition within Au@CeO_2_ composition a; their findings revealed that Au@CeO_2_ has superoxide dismutase (SOD) mimetic enzyme activity, which can effectively inhibit cell cycle progression and proliferation in acute myelogenous leukemia [20]. Hussain group has developed a series of Ag_2_O/Ce_2_O that has a p–n heterostructure via a facile hydrothermal approach. The crystal structure, morphology, chemical composition, optical properties, and photocatalytic activity were investigated to obtain high photocatalytic efficiency material [21]. It is well known that Ag nanoparticles have high antibacterial activity and antioxidant properties, which offer important applications including antibacterial treatment, immunomodulation, burn care, and skin repair [22,23]. Additionally, Ag nanoparticles have been successfully employed for drug delivery and cancer therapy due to their high electrical conductivity, excellent thermal conductivity, chemical stability, catalytic activity, and enhanced Raman scattering properties [24,25]. Ag@CeO_2_ composite nanoparticles represent an innovative approach in this field.

In this work, we aimed to synthesize Ag@CeO_2_ nanoparticles, which possess significant antitumor properties and are capable of serving as a drug in ECT to potentially enhance their therapeutic efficacy. In a proof-of-concept study, we developed three distinct synthesis methods for Ag@CeO_2_ and characterization by TEM, XRD, SEM, BET, FITR, UV-vis, XPS, TGA, and EIS and comprehensively evaluated their antioxidant, cytotoxic, and antitumor activities. Ag@CeO_2_ ECT-induced changes in cellular activity, redox system, and mitochondrial membrane potential were also investigated. Our findings suggest that Ag@CeO_2_ is a feasible candidate for an ECT drug, offering the potential for targeted treatment of breast cancer.

## 2. Materials and Methods

### 2.1. Chemicals and Materials

Ammonia (25 wt.%), cerium nitrate hexahydrate (Ce(NO_3_)_3_·6H_2_O), silver nitrate (AgNO_3_), and urea were purchased from Sinopharm Reagents Co., Ltd. (Shanghai, China). Reduced glutathione (GSH) and DMSO were purchased from Shanghai Sigma-Aldrich Co., Ltd. (Shanghai, China). Hydrogen peroxide (H_2_O_2_, 30 wt.%) was purchased from Shanghai Aladdin Biochemical Technology Co., Ltd. (Shanghai, China). Cell proliferation and cytotoxicity assay kit (CCK-8), PBS buffer (pH = 7.4), bovine serum protein (BSA), fetal bovine serum (FBS), and trypsin were purchased from Beijing Solaibao Biotechnology Co., Ltd. (Beijing, China).

### 2.2. Synthesis and Characterization of CeO_2_ and Ag@CeO_2_ Nanoparticles

CeO_2_ synthesis: Ammonia solution (25 wt%) was gradually added to a Ce(NO_3_)_3_ solution (2 g, 20 mL) with pH = 12. The precipitate was washed three times with deionized water (H_2_O) followed by centrifugation at 7104 g for 4 min until the supernatant was achieving neutral supernatant conditions. The precipitate was then dried overnight in an oven set at 80 °C before being calcined in a muffle furnace at a temperature of 500 °C for 5 h.

Ag@CeO_2_-1 co-precipitation synthesis: Aqueous ammonia solution (25 wt%) was slowly added dropwise to an aqueous mixture containing 5.05 g Ce(NO_3_)_3_·6H_2_O and 0.265 g AgNO_3_ while stirring continuously, and this solution was adjusted to reach pH = 12. Subsequently, the mixture underwent centrifugation at 7104 g for 4 min. The precipitate obtained was washed three times with deionized water until achieving clarity in a supernatant neutral. After that, the precipitate was dried overnight in an oven at 80 °C and calcined in a muffle furnace at 500 °C for 5 h.

Ag@CeO_2_-2 homogeneous precipitation synthesis: A total of 10 g of urea was dissolved in 30 mL of deionized water with vigorous stirring at room temperature. Subsequently, a mixture consisting of 2.6 g of Ce(NO_3_)_2_·6H_2_O and 0.08 g AgNO_3_ was added while maintaining vigorous stirring for 2 h to obtain a clear solution. This solution was then dried in an oven at 70 °C under forced air circulation for 24 h. Following this, the resulting material was calcined in a muffle furnace at 500 °C.

Ag@CeO_2_-3 post-impregnation synthesis: An amount of 1 g from the prepared CeO_2_ was taken and combined with 8.0 mL of AgNO_3_ solution, followed by stirring for approximately 5 h. The resultant solution was dried overnight in an oven set to a temperature of 60 °C and subsequently calcined in a muffle furnace at 500 °C for a period of three hours.

This study focuses on enhancing selective tumor cell targeting and reducing non-specific uptake by normal cells to improve the therapeutic index of Ag-CeO_2_ composition in oncology. The size distribution of three synthesized Ag-CeO_2_ formulations was optimized via differential centrifugation following 30 min of ultrasonication (power 100 W). Ag-CeO_2_ nanoparticles exceeding 50 nm in diameter were isolated through step-gradient centrifugation at forces ranging from 3025 g to 12,100 g for 20 min to minimize off-target effects. The collected precipitates were vacuum-dried at 60 °C for 6 h, cooled to room temperature, and stored for subsequent experiments. To evaluate stability and dispersion kinetics in physiological conditions, dynamic light scattering (DLS) measurements were performed on Ag-CeO_2_ suspensions in DMEM culture medium. Samples were analyzed after quiescent incubation for 2–12 h post-dispersion, with ultrapure water serving as a blank control.

### 2.3. Cell Culture and Characterization

MCF-7 cells were cultured in Dulbecco’s Modified Eagle Medium (DMEM) supplemented with 10% fetal bovine serum (FBS) and 1% penicillin-streptomycin mixture, under a controlled atmosphere of 5% CO_2_ at 37 °C. Following, the MCF-7 cells were subjected to enzymatic digestion with trypsin, and detached from the Petri dishes and then underwent centrifugation by 1000 rpm for 3 min. The CCK-8 method and Calcein AM/PI cell viability assay kit were employed to assess cell proliferation and viability. Reactive oxygen species (ROS), glutathione levels, apoptosis, and cell cycle distribution were analyzed utilizing DCFH-DA, 2,3-naphthalene formaldehyde (NDA), and Annexin V-FITC/PI apoptosis detection kit, and cell apoptosis and cell cycle phases were examined by flow cytometry. Fluorescence images were captured using confocal laser scanning microscopy (CLSM) at wavelengths of 488 nm and 552 nm, while the intensity was quantified through ImageJ 1.51j8 software. Additionally, intracellular mitochondrial membrane potential was assessed via JC-1 probe assay.

### 2.4. Antioxidant and Cellular Electrochemotherapy of CeO_2_ and Ag@CeO_2_

A total of 100 μL 2,2′-bis (3-ethylbenzothiazoline-6-sulfonic acid) diammonium salt (ABTS, 3.84 g/mL) and 100 μL H_2_O_2_ (0.1 μM) were mixed in each 96-well plate and then added to 10 μL of PBS solution as a blank control. Adding 10 μL of peroxidase was as a control experiment. An amount of 10 μL 1 mg/mL CeO_2_ or Ag-CeO_2_ samples was added to every detection well to study their antioxidant properties. After incubation at room temperature for 6 min, the absorbance values of each well at 435 nm were measured in an enzyme-linked immunosorbent assay (ELISA) reader. Cellular electrochemotherapy was taken under 2 V because 2 V will be used as the electroporation voltage in all the next ECT experiments, and 0 V will also be set as the control. Ag@CeO_2_ concentration in ECT was optimized by cellular experiments.

## 3. Results and Discussion

### 3.1. Characterization of Ag@CeO_2_

**Figure 1:**
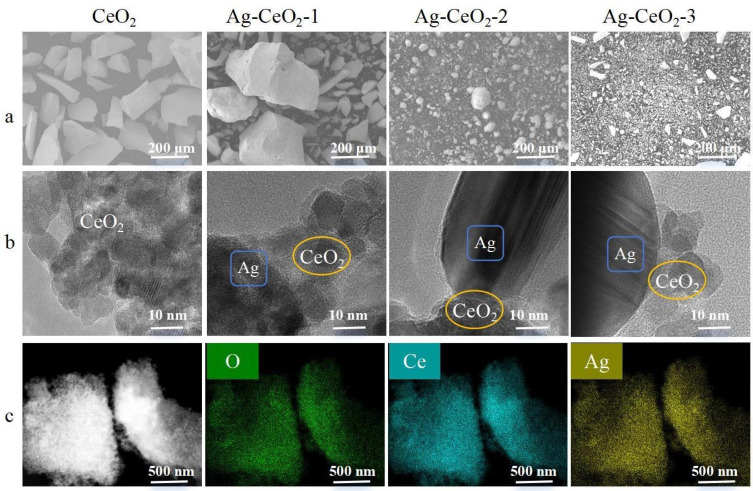
Morphological and compositional characterization of CeO_2_ and Ag@CeO_2_ nanocomposites. (**a**) SEM images showing agglomerated CeO_2_ particles in Ag@CeO_2_-1 (left) and well-dispersed morphologies in Ag@CeO_2_-2 (middle) and Ag@CeO_2_-3 (right). (**b**) TEM images revealing the proximity of Ag nanoparticles (spherical and flat) to CeO_2_ matrices (polyhedral shape with a rough surface). (**c**) Elemental mapping (EDS) of Ag@CeO_2_ nanocomposites, demonstrating co-localization of Ag (yellow) and Ce (blue) signals, confirming successful composite formation rather than physical mixture.

The SEM characterization of the synthesized Ag@CeO_2_ in Figure 1a revealed significant agglomerations of CeO_2_ particles in Ag@CeO_2_-1, whereas the Ag@CeO_2_-2 and Ag@CeO_2_-3 particles obtained exhibited a more dispersed morphology. Further TEM characterization in Figure 1b facilitated a clearer observation of the particle morphology of the synthesized nanoparticles. Combined with mapping elemental analysis in Figure 1c, it confirmed the presence of both Ag and CeO_2_ of synthesized Ag@CeO_2_ nanoparticles, indicating the successful preparation of Ag@CeO_2_ nanoparticles.

**Figure 2:**
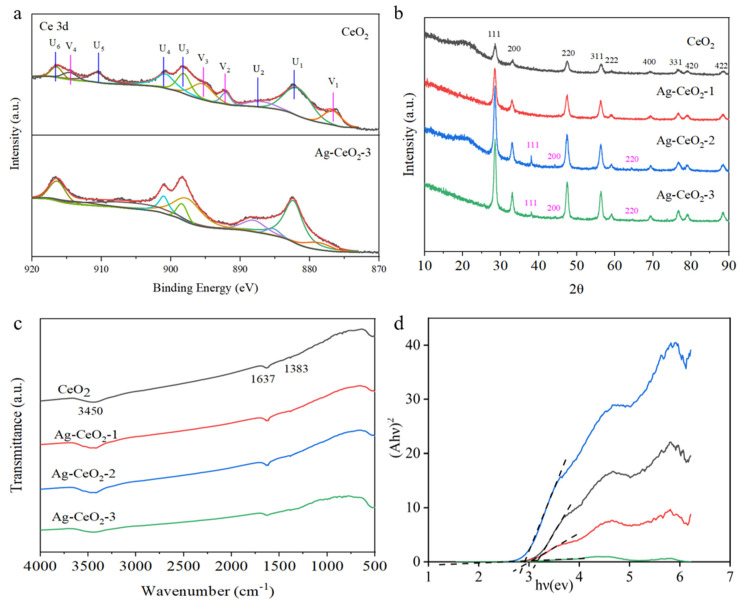
Characterization of CeO_2_ and Ag@CeO_2_: (**a**) XPS analysis, (**b**) XRD, (**c**) FITR, (**d**) Tauc.

XPS analysis in Figure 2a presents Ce 3d in both pure CeO_2_ and Ag@CeO_2_-3 samples. A comparison between these two samples reveals that the Ce 3d spectrum displays a rather complex structure. The peaks labeled U1–U6 correspond to six peaks associated with Ce^4+^ 3d (881.73, 886.95, 895.45, 900.76, 910.61, and 916.47 eV), while V1–V4 represent four peaks related to Ce^3+^ 3d (876.69, 892.09, 893.13, and 914.57 eV). It is calculated that the percentage of Ce^4+^ in pure CeO_2_ is approximately 71.34%, while that for Ce^3+^ is about 28.66%. With the addition of Ag, it can be determined that the percentage of Ce^4+^ in Ag@CeO_2_-3 was increased to approximately 85.35%, confirming electronic interactions between Ag and CeO_2_, while the percentage of Ce^3+^ decreases to around 14.65%. This indicates that incorporating Ag promotes an increase in the proportion of Ce^4+^, thereby enhancing enzyme mimetic activity, which favors biological applications.

Structural characterization conducted via XRD demonstrated that pure CeO_2_ exhibits a classical crystalline fluorite structure characterized by several distinct diffraction peaks at angles including 2θ = 28.5°, 33.1°, 47.5°, 56.3°, 59.1°, 69.4°, 76.7°, 79.1°, and 88.4° in Figure 2b. These peaks correspond to the (111), (200), (220), (311), (222), (400), (331), (420), (422) planes in JCPDS card No. 34-0394 and (311), (222), (400), (331), (420), (422) planes, respectively, as listed in JCPDS card No. 34-0394. The Ag@CeO_2_ nanoparticles synthesized using method I show almost no observed peaks corresponding to metallic silver, indicating that Ag is highly dispersed on the surface of CeO_2_, making it difficult to detect. In contrast, XRD analysis of Ag@CeO_2_-2 and Ag@CeO_2_-3 revealed peaks at 2θ = 38.2°, 44.2°, and 68.4°, corresponding to the (111), (200), and (220) planes of face-centered cubic Ag (JCPDS File No. 89-3722), corresponding to the (111), (200), and (220) planes, associated with Ag composition, respectively (JCPDS File No. 89-3722). These results indicated that Ag had been successfully loaded onto the CeO_2_ surface.

FTIR spectra of pure CeO_2_, Ag@CeO_2_-1, Ag@CeO_2_-2, and Ag@CeO_2_-3 were characterized in Figure 2c. The broad peak observed at 3450 cm^−1^ is attributed to the stretching vibrations of the O-H group, likely resulting from moisture absorbed from the air onto the sample surfaces. The absorption band at 1637 cm^−1^ corresponds to an O-Ce-O bond and resembles the bending mode of H-O-H. A minor peak at 1384 cm^−1^ can be associated with the bending vibrations of Ce-O bonds, while the absorption peak detected at 554 cm^−1^ relates to Ce-O bond vibrations. Notably, the peaks for both pure CeO_2_ and variously prepared Ag@CeO_2_ nanoparticles remained nearly identical. Moreover, the high dispersion of Ag loaded onto CeO_2_ surfaces made it difficult to observe Ag by FTIR. As shown in Figure 2d, the band gap of CeO_2_, Ag-CeO_2_-1, Ag-CeO_2_-2, and Ag-CeO_2_-3 is about 3.14 eV, 3.01 eV, 2.93 eV, and 2.67 eV, respectively. The band gap energy decreased when Ag was coupled with CeO_2_, and the Ag-CeO_2_ nanoparticles with low band gap energy exhibited better performance in visible light-induced reactions. This also proves that the plasmon resonance effect of the precious metal Ag caused the formation of localized energy levels within the original CeO_2_ band gap, thereby reducing band gap.

**Figure 3:**
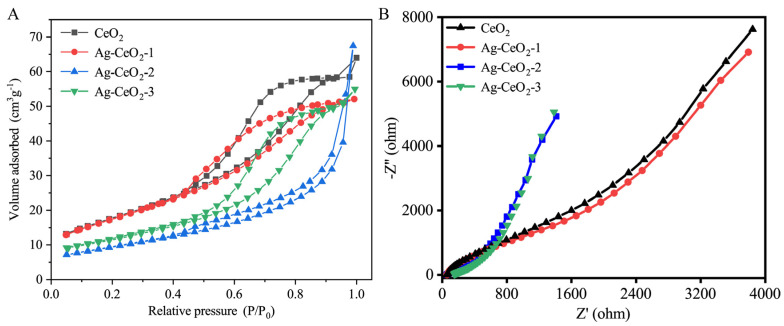
Characterization of CeO_2_ and Ag@CeO_2_: (**A**) BET analysis, (**B**) EIS.

Figure 3A presents the N_2_ adsorption-desorption isotherms of pure CeO_2_ and Ag@CeO_2_ composite nanomaterials. CeO_2_, Ag@CeO_2_-1, and Ag@CeO_2_-3 displayed type IV isotherms with H3 hysteresis loops, likely resulting from the aggregation of cubic particles and the subsequent formation of slit-like pore structures. In contrast, Ag@CeO_2_-2 showed typical type IV isotherms with H2 hysteresis loops, which may stem from pores formed by composition agglomeration. Slit porous materials are well-known for their significant role in biocontainment, as drug molecules can be easily transported and loaded within the porous structure. The pore size and specific surface area of the samples were determined from the pore size distribution curves in Table 1. The specific surface areas of CeO_2_, Ag@CeO_2_-1, Ag@CeO_2_-2, and Ag@CeO_2_-3 are 64.5235 m^2^/g, 63.4621 m^2^/g, 33.9442 m^2^/g, and 41.3521 m^2^/g, respectively. While N_2_ adsorption-desorption isotherms and pore size distribution provide critical insights into the textural properties of the materials, it should be noted that these data primarily reflect bulk structural features rather than directly proving silver loading. The observed differences in hysteresis loops (H3 for Ag@CeO_2_-1/3 vs. H2 for Ag@CeO_2_-2) and specific surface area reduction (from 64.52 m^2^/g for pure CeO_2_ to 41.35 m^2^/g for Ag@CeO_2_-3) are consistent with silver incorporation but require validation through complementary techniques. Furthermore, XPS analysis in Figure 2a demonstrated altered Ce^3+^/Ce^4+^ ratios, confirming electronic interactions between Ag and CeO_2_. XRD patterns in Figure 2b revealed distinct metallic Ag peaks (2θ = 38.2°, 44.2°) in Ag@CeO_2_-2/Ag@CeO_2_-3, absent in pure CeO_2_. These combined results provide unambiguous evidence for successful silver loading, with N_2_ isotherms serving as secondary indicators of structural modification.

Figure 3B presents electrochemical impedance spectroscopy (EIS) analysis of the prepared samples, employed to assess their electron transfer efficiency. A smaller semicircle diameter in the Nyquist plot signifies lower resistance. The Ag@CeO_2_ nanoparticles exhibited markedly reduced resistance compared to pure CeO_2_, notably Ag@CeO_2_-2, which exhibits the smallest charge transfer resistance. It is speculated that silver (Ag), being a good conductor, forms a conductive network when doped into cerium dioxide, thereby improving electronic conductivity. Furthermore, optimized dispersion in Ag@CeO_2_-2 may contribute to its superior conductivity. Besides, the sharp increase in the slope of the straight line for Ag@CeO_2_-2 and Ag@CeO_2_-3, indicating that these materials of Ag@CeO_2_-2 and Ag@CeO_2_-3 are primarily electron conductive, is good electronic conductivity. The introduction of silver significantly improved the conductivity of ceria, and with the optimization of doping conditions, the conductivity was further enhanced. This indicates significantly enhanced interfacial photogenerated charge transfer efficiency, attributed to Ag composition loading. This advantageous electrical property not only boosts electron transfer but also promises to minimize external environmental interference in cell impedance detection, making Ag@CeO_2_-3 a promising candidate for high-sensitivity biosensing applications.

From Figure 3, strong interfacial bonding (Ce-O-Ag) of Ag@CeO_2_-1 promotes electron transfer from Ag to CeO_2_, increasing the proportion of Ce^3^⁺ and the concentration of oxygen vacancies, while Ag exists in an oxidized state, enhancing the interfacial charge transfer capacity with low impedance. Ag@CeO_2_-2 presents Ag particles that are in situ dispersed on the surface of porous CeO_2_ in a metallic state of Ag^0^, with a moderate concentration of oxygen vacancies and interfacial contact, resulting in moderate impedance. For Ag@CeO_2_-3, there is only physical adsorption between Ag and CeO_2_, with hindered interfacial electron transfer and high impedance, and Ag exists as large Ag^0^ particles with a low proportion of Ce^3^⁺. Besides, the interfaces of Ce-O-Ag in Ag@CeO_2_-1 promote the transfer of electrons from Ag to CeO_2_, increasing the proportion of Ce^3+^ and leading to more oxygen vacancies, while Ag exists in an oxidation state that enhances the charge transfer ability of the interface of low impedance. Ag particles in Ag@CeO_2_-2 are dispersed in situ on the surface of porous CeO_2_ in a metallic state (Ag^0^), with moderate oxygen vacancy concentration and interfacial contact. Ag@CeO₂-3 exhibits physical adsorption between Ag and CeO₂, the interface electron transfer is blocked, and Ag exists in large particle Ag^0^ and a low proportion of Ce^3+^. As a result, Ag@CeO_2_ materials synthesized by three different methods have different oxygen vacancies and, consequently, different antioxidant abilities.

In short, three synthetic methods were employed to investigate the distinct binding mechanisms between Ag nanoparticles and CeO_2_. Ag@CeO_2_-1 was synthesized via co-precipitation under strongly alkaline conditions, forming an Ag-Ce composite hydroxide precursor. After calcination, Ag was embedded into the CeO_2_ lattice, inhibiting its growth. However, lattice distortion caused broadening of the XRD peaks. Ag@CeO_2_-2 was prepared using homogeneous precipitation with urea to slowly release NH_3_, forming a homogeneous precursor. Low-temperature drying preserved a porous structure, and subsequent calcination yielded small crystallite CeO_2_ with uniformly dispersed metallic Ag^0^ (high specific surface area). Ag@CeO_2_-1 was fabricated via impregnation, physically adsorbing Ag onto pre-formed CeO_2_ surfaces. During calcination, Ag migrated and agglomerated into large Ag^0^ particles (distinct metallic Ag peak), leading to pore blockage (lowest specific surface area), hindered interfacial electron transfer (high impedance), and a reduced proportion of Ce^3+^. The three methods resulted in distinct Ag-support interactions: lattice embedding, uniform dispersion, and surface agglomeration, respectively.

**Figure 4:**
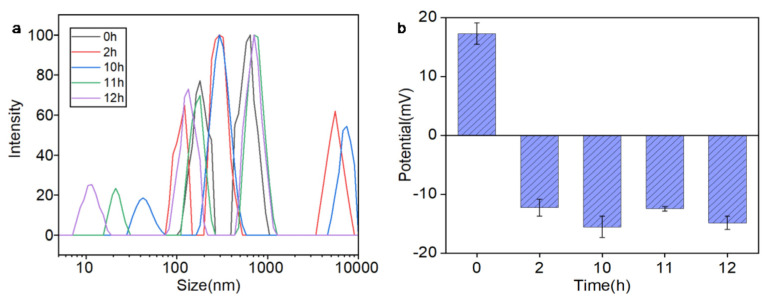
Stability profile of Ag@CeO_2_-3 nanoparticles in DMEM + 10% FBS: (**a**) The relationship between size and intensity; (**b**) Zeta potential transition from +17 mV (0 h) to −15 mV (2–12 h) due to protein corona formation. The shaded region (0–10 h) indicates the optimal window for cellular uptake, during which nanoparticles maintain sufficient dispersion for biological activity. All cellular experiments were conducted within 24 h to ensure nanoparticle functionality.

**Figure 5:**
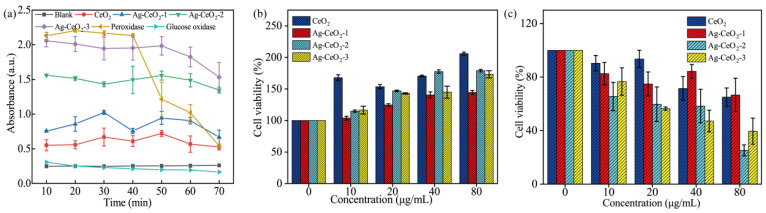
Characterization of CeO_2_ and Ag@CeO_2_: (**a**) antioxidant activity, (**b**) differential cytotoxicity observed between HUVEC (**b**) and MCF-7cells (**c**) after 24 h exposure.

**Figure 6:**
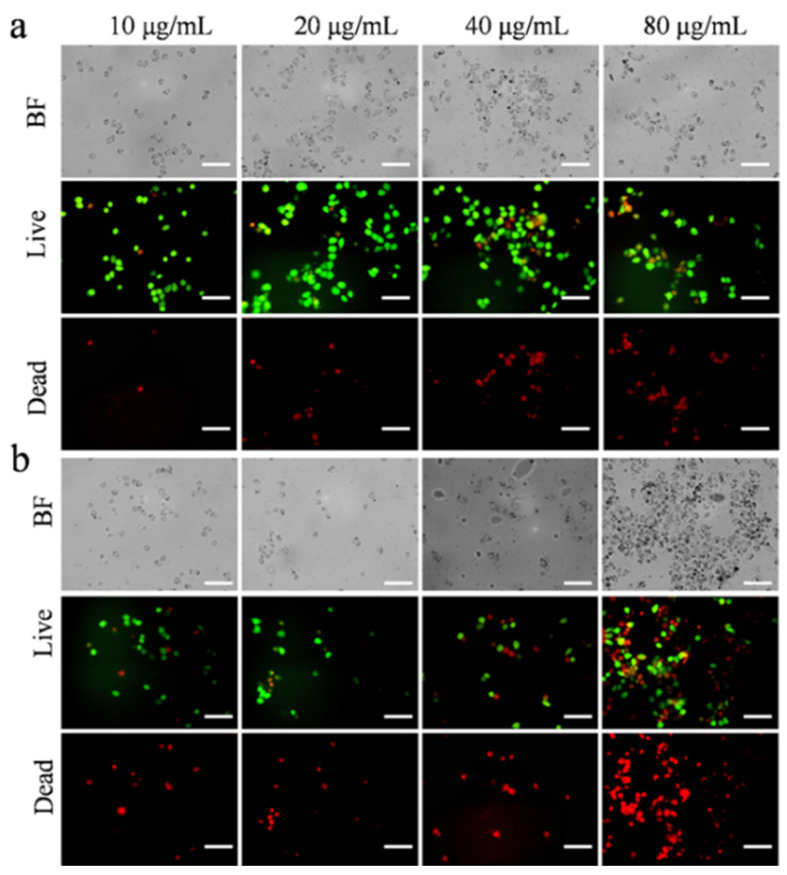
The distribution of live/dead MCF-7 cells after 24 h treatment by (**a**) different concentrations of CeO_2_, (**b**) by different concentrations of Ag@CeO_2_. Scale bar is 100 μm.

**Figure 7:**
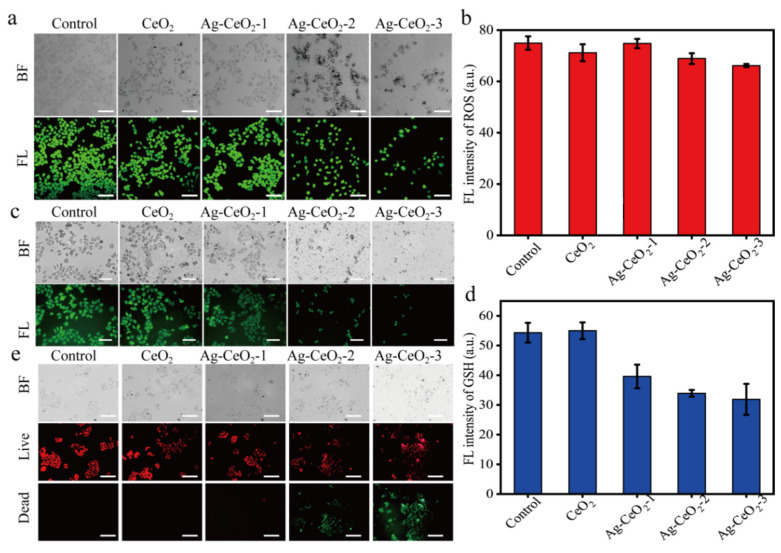
Intracellular redox reactions of MCF-7 cells by treatment with CeO_2_, Ag@CeO_2_-1, Ag@CeO_2_-2, and Ag@CeO_2_-3 for 24 h. (**a**) Changes in ROS content. (**b**) Corresponding fluorescence intensity analysis of ROS. (**c**) Changes in GSH content. (**d**) Corresponding fluorescence intensity analysis of GSH. (**e**) Changes in mitochondrial membrane potential. Scale bar is 100 μm.

DLS and zeta potential analyses in Figure 4 revealed a time-dependent stability profile of Ag@CeO_2–3_ nanoparticles in biological media. Initially, the nanoparticles exhibited a positive zeta potential (+17 mV) and moderate hydrodynamic size (~140 nm), favoring electrostatic interactions with negatively charged cell membranes and facilitating early cellular uptake (0–2 h). However, within 2 h, the zeta potential shifts to negative values (−15 mV) due to protein corona formation, coinciding with a gradual increase in particle size (reaching ~200 nm by 12 h). This transition confirmed progressive aggregation, consistent with colloidal instability at low absolute zeta potentials (<30 mV). Critically, the nanoparticles maintained partial dispersion and bioactivity during the first 10 h in Figure 4a, providing a sufficient temporal window for cellular internalization in our biological assays (conducted within 24 h). The initial positive charge and nanoscale size (30–60 nm) enabled selective tumor cell targeting in Figure 5, Figure 6 and Figure 7, while subsequent aggregation limits long-term stability but did not preclude therapeutic efficacy in short-term treatments. These findings underscore the importance of timing in nanoparticle-based therapies, where early cellular uptake precedes significant aggregation-mediated loss of function.

The observed differential cytotoxicity between tumor and normal cells suggests time-dependent selective effects mediated by the initial nanoparticle properties (0–10 h window), though material stability limitations must be acknowledged. While the initial positive zeta potential (+17 mV) may facilitate tumor cell interactions, the aggregation behavior precludes definitive conclusions about long-term targeting specificity. This selective targeting is further supported by the nanoparticles’ ability to maintain moderate dispersion in the medium for up to 10 h, allowing sufficient time for tumor cell interaction before aggregation occurs. The progressive aggregation and charge reversal observed after 11 h may reduce the nanoparticles’ targeting efficacy, highlighting the importance of optimizing the time frame for their application in biological environments to maximize therapeutic benefits while minimizing off-target effects. Additionally, the nanoparticles’ antioxidant properties and their ability to modulate the tumor microenvironment could contribute to their selective effects on tumor cells, as they may protect normal cells from oxidative damage while exerting cytotoxic effects on tumor cells through mechanisms such as H_2_O_2_ accumulation and Ce^4+^ ion release in the acidic tumor microenvironment.

### 3.2. Evidence for Ag Loading and Composite Formation

The successful loading of Ag nanoparticles onto CeO_2_ and the formation of Ag@CeO_2_ nanocomposites were systematically verified through multiple characterization techniques. XPS revealed a significant increase in the Ce^4+^/Ce^3+^ ratio (from 71.34% to 85.35%) in Ag@CeO_2_-3 in Figure 2a, indicating electron transfer from Ag to CeO_2_ and confirming strong interfacial interactions. XRD patterns in Figure 2b exhibited distinct peaks for metallic Ag (2θ = 38.2°, 44.2°, and 68.4°) in Ag@CeO_2_-2 and Ag@CeO_2_-3, confirming crystalline Ag formation, while their absence in Ag@CeO_2_-1 suggested highly dispersed Ag species. TEM coupled with elemental mapping in Figure 1c demonstrated the co-localization of Ag and Ce, ruling out mere physical mixing. N_2_ adsorption-desorption isotherms in Figure 3A and pore size distribution analysis further supported composite formation: Ag@CeO_2_-1 and Ag@CeO_2_-3 displayed slit-like pores (H3 hysteresis), whereas Ag@CeO_2_-2 showed uniform mesopores (H2 hysteresis), correlating with synthesis-dependent Ag dispersion. Additionally, EIS in Figure 3B revealed reduced charge transfer resistance in Ag@CeO_2_-2, attributed to optimized Ag-CeO_2_ interfaces. Collectively, these results provide unambiguous evidence for the successful synthesis of Ag@CeO_2_ nanocomposites with distinct structural and electronic properties.

### 3.3. Antioxidant and Cytotoxicity of CeO_2_ and Ag@CeO_2_

Although the role of antioxidants in tumor treatment remains controversial, they can serve as adjuvant therapies to alleviate oxidative stress damage during treatment, thereby enhancing treatment efficacy. For instance, by modulating the redox status in the tumor microenvironment, the effectiveness of chemotherapy and radiotherapy can be augmented. Future tumor treatments could be personalized based on the stage of the tumor, the redox status of tumor cells, and the individual immune function of patients. This personalized approach can help maximize the potential benefits of antioxidants while mitigating their potential risks. Peroxidase, a well-known free radical scavenger, is one of the most traditional antioxidant marker enzymes. Therefore, the ABTS-H_2_O_2_ system was employed to evaluate the antioxidant activity of CeO_2_ and Ag@CeO_2_. As shown in Figure 5a, both CeO_2_ and Ag@CeO_2_ exhibit peroxidase activity and possess high antioxidant stability, making them promising candidates for sustained drug release. Notably, Ag@CeO_2_-3 demonstrates the highest peroxidase activity [26], showing the strongest antioxidant activity, comparable to that of peroxidase and with a longer-lasting effect, suggesting its potential use in free radical scavenging drugs and tumor antioxidant therapy. These results are consistent with the XPS and EIS analysis results that Ag@CeO_2_-3 has highest antioxidant abilities. Furthermore, antioxidants are typically used to neutralize free radicals, which are harmful to cells. Therefore, antioxidant materials may help reduce cell damage and lower cytotoxicity. In this work, Ag@CeO_2_ materials are composite materials composed of silver and CeO_2_. CeO_2_ itself is a commonly used antioxidant material, especially nano-cerium dioxide, because it has a Ce^3+^/Ce^4+^ redox cycle, which can eliminate free radicals. Ag nanoparticles have antibacterial properties, but they may also have cytotoxicity. The combination of the two may produce synergistic or antagonistic effects. Hence, the cytotoxicity of both CeO_2_ and Ag@CeO_2_ is investigated, and they are expected to act as sustained-release drugs, which were evaluated using the CCK-8 kit in the exposure of MCF-7 cancer cells as well as normal HUVEC cells for 24 h. As illustrated in Figure 5b,c, Ag@CeO_2_-3 demonstrated time-dependent selective cytotoxicity, with >60% cell death in MCF-7 versus <20% in HUVECs at 80 μg/mL during the 24 h treatment window (Figure 5b,c), suggesting differential cellular responses under these experimental conditions, which promotes tumor cell death but has little effect on normal endothelial cells. This is due to the sizes of the prepared material being between 30 and 60 nm. Of this size, normal cells have less endocytosis, reduce non-specific uptake, and reduce toxicity. Moreover, consider the differences between tumor cells and normal cells. MCF-7 cells typically have higher basal levels of ROS, making them more sensitive to oxidative stress. HUVEC cells have lower ROS levels and a well-balanced antioxidant defense system. Therefore, the antioxidant effect of Ag@CeO_2_ may help maintain oxidative balance in HUVEC cells, while in MCF-7 cells, it may promote cell death by further regulating ROS levels. Ag@CeO_2_-3 have the highest antioxidant activity, modulating the redox system in the tumor microenvironment, thus enhanced antitumor activity.

To further understand the high cytotoxicity of Ag@CeO_2_ on tumor cells but little effect on HUVEC cells, cell viability after exposure on the synthesized cerium-based nanomaterials was investigated by using a live/dead cell double staining kit (Calcein-AM and PI kit) (Beyotime Biotechnology, Shanghai, China). Viable cells appeared green, while dead cells displayed red fluorescence. MCF-7 cells treated with Ag@CeO_2_ demonstrated increased red fluorescence indicative of higher levels of cell death. In addition, cell apoptosis was enhanced along with cellular shrinkage. Bright field imaging revealed that the introduced Ag@CeO_2_ nanoparticles either entered or adhered more readily to the surface of tumor cells, providing preliminary evidence that Ag@CeO_2_ nanoparticles are preferentially internalized by tumor cells, leading to substantial accumulation within them. Ag@CeO_2_ includes reversible Ce^3+^/Ce^4+^ redox pairs in the lattice of CeO_2_, which can clear ROS such as superoxide radicals (O^2−^) and hydroxyl radicals (OH^−^), through surface oxygen vacancies. This finding offers valuable insights for subsequent applications in drug treatment or electrochemotherapy. As depicted in Figure 6, CeO_2_ induced tumor cell death in a small fraction of treated cells. Concurrently, it was observed that as concentrations of Ag@CeO_2_ increased, there was a corresponding rise in dead cell proportions of MCF-7 cells. In comparison to CeO_2_ alone, the presence of Ag@CeO_2_ resulted in markedly greater cytotoxicity, culminating in extensive cell death—particularly pronounced when sample concentrations reached 80 μg/mL. These findings further substantiate that while CeO_2_ composition exhibits lower toxicity towards cancerous tissues, Ag@CeO_2_ exhibits higher toxicity towards cancerous tissues. These findings demonstrate that the antioxidant properties of Ag@CeO_2_ compete dynamically with its cytotoxicity, regulated by the amount of silver loading. At low concentrations, protective effects are observed: Ag@CeO_2_ neutralizes ROS through the redox cycle of CeO_2_, alleviating oxidative stress and protecting cells from DNA damage and apoptosis. Low concentrations of Ag^+^ inhibit the growth of pathogens, reducing inflammation caused by infection and indirectly lowering cell toxicity. At high concentrations of Ag@CeO_2_, excessive Ag^+^ release would lead to mitochondrial membrane potential collapse, inhibition of ATP synthesis, and activation of the pathway, causing cell apoptosis or necrosis. Moreover, ROS clearance ability of CeO_2_ may be exhausted in high ROS environments, leading to Ag’s toxicity becoming dominant.

### 3.4. Changes in Intracellular Redox System by Using CeO_2_ and Ag@CeO_2_

ROS and GSH are two critical indicators of the cellular redox system. DCFH-DA itself is non-fluorescent. However, it can readily penetrate the cell membrane, where it undergoes enzymatic cleavage to yield DCFH, which cannot traverse the cell membrane. The generated ROS can oxidize the non-fluorescent DCFH into fluorescent DCF, resulting in green fluorescence within the cells. As shown in Figure 7a,b, all cells exhibited strong green fluorescence. Notably, the introduction of Ag@CeO_2_-2 and Ag@CeO_2_-3 induced cell death and led to cellular abscission. Furthermore, the data of GSH fluorescence displayed a different trend compared to that of ROS in Figure 7c,d, indicating that the uptake capacity of Ag@CeO_2_-2 and Ag@CeO_2_-3 was significantly greater than that of CeO_2_ and Ag@CeO_2_-1.

To further elucidate the intracellular redox effects of synthesized CeO_2_ and Ag@CeO_2_ on tumor cells, we employed a mitochondrial membrane potential assay kit (JC-1) to assess changes in mitochondrial membrane potential. The results revealed that the addition of CeO_2_ or Ag@CeO_2_ may be contributing to elevated intracellular ROS levels, subsequently disrupting mitochondrial membranes and altering cell membrane permeability. This disruption allowed ions inside and outside the cell membrane to equilibrate through free diffusion, diminishing both intra- and extracellular concentration gradients and leading to a reduction in membrane potential (evidenced by green fluorescence), thereby exacerbating ROS production. As shown in Figure 7e, tumor cells treated with Ag@CeO_2_-2 and Ag@CeO_2_-3 have a significant decrease in viability, further demonstrating that the expolite of Ag@CeO_2_ greatly changes the intracellular redox of tumor cells, which results in the mitochondrial membrane potential, and then exhibits higher toxicity towards cancerous tissues.

### 3.5. Ag@CeO_2_ Based ECT

**Figure 8:**
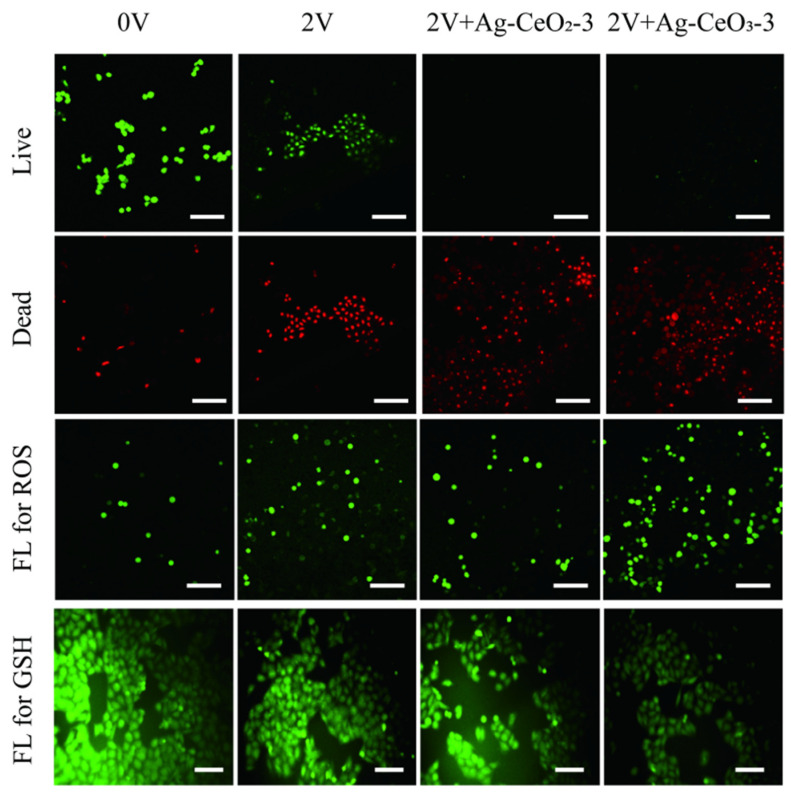
Cell live/dead, ROS and GSH of MCF-7 cells after treatment with 0 V, 2 V, 2 V + Ag@CeO_2_-2, 2 V + Ag@CeO_2_-3. Scale bar is 100 μm.

The high antioxidant and anti-tumor activity of Ag@CeO_2_-2 and Ag@CeO_2_-3 were further investigated concerning ECT. A stimulation voltage was set at 2 V due to its significant impact on cell activity as reported previously [27]. As illustrated in Figure 8, under a voltage stimulation of 2 V, a marked reduction in cell viability was observed compared to the control group. When combining this voltage stimulation with either 40 μg/mL Ag@CeO_2_-2 or Ag@CeO_2_-3, there was a substantial increase in dead cells noted. This indicates that both Ag@CeO_2_-2 and Ag@CeO_2_-3 are effective agents for tumor ECT, presenting promising alternatives to conventional adriamycin therapies. It can be deduced that when subjected to voltage stimulation, cell membranes become permeable; consequently, Ag@CeO_2_ enters the cells through an opening created in the membrane, resulting in significant accumulation within various cellular compartments. This accumulation leads to an imbalance in intracellular ROS production and causes depolarization of mitochondrial transmembrane potential, accompanied by excessive ROS generated along with decreased GSH expression. The data in Figure 8 confirmed our speculation. Furthermore, the high levels of tumor cell death and enhanced anti-tumor efficacy indicate that it could serve as a potent candidate for ECT along with its introduction into therapeutic protocols.

## 4. Conclusions

In summary, this study successfully synthesized and characterized silver-decorated cerium oxide (Ag@CeO_2_) nanoparticles, demonstrating their enhanced antioxidant and cytotoxic properties. The high antioxidant activity and tumor-specific cytotoxicity of Ag@CeO_2_ highlight its potential as a promising alternative to conventional tumor treatments in ECT. The results revealed that Ag@CeO_2_ nanoparticles, particularly Ag@CeO_2_-2 and Ag@CeO_2_-3, induced significant cell death in MCF-7 tumor cells under electrical stimulation at 2 V, achieving effective therapeutic outcomes. Furthermore, ECT-induced changes in cell morphology and intracellular redox dynamics suggest that the mechanism involves the excessive production of ROS triggered by Ag@CeO_2_. This ROS overproduction likely damages mitochondria and other critical cellular structures, accelerating cellular injury and promoting tumor cell death. However, further research is needed to fully elucidate the underlying mechanisms and optimize the therapeutic potential of Ag@CeO_2_ nanoparticles in ECT. Moreover, this study has several limitations: (1) the physical adsorption synthesis yields some heterogeneity in nanoparticle composition; (2) the time-dependent aggregation requires careful control of experimental timing; and (3) the selective effects are demonstrated under specific in vitro conditions that may not directly translate to in vivo applications. These findings underscore the significant promise of Ag@CeO_2_ in advancing material-based tumor treatment strategies.

## Figures and Tables

**Table 1:** Surface area, volume, and pore size of different nanomaterials.

Sample Name	SBET (m^2^/g)	Pore Volume (cm^3^/g)	Pore Size (nm)
CeO_2_	64.5235	0.0904	5.6067
Ag@CeO_2_-1	63.4621	0.0800	5.0437
Ag@CeO_2_-2	33.9442	0.0875	10.3162
Ag@CeO_2_-3	41.3521	0.0817	7.9067

## Data Availability

No data were used for the research described in the article.
